# Devaluation of Unchosen Options: A Bayesian Account of the Provenance and Maintenance of Overly Optimistic Expectations

**Published:** 2020

**Authors:** Corey Yishan Zhou, Dalin Guo, Angela J. Yu

**Affiliations:** Department of Cognitive Science, University of California, San Diego La Jolla, CA 92093 USA

**Keywords:** unrealistic optimism, decision making, multi-armed bandit, reinforcement learning, Bayesian modeling

## Abstract

Humans frequently overestimate the likelihood of desirable events while underestimating the likelihood of undesirable ones: a phenomenon known as *unrealistic optimism*. Previously, it was suggested that unrealistic optimism arises from asymmetric belief updating, with a relatively reduced coding of undesirable information. Prior studies have shown that a reinforcement learning (RL) model with asymmetric learning rates (greater for a positive prediction error than a negative prediction error) could account for unrealistic optimism in a bandit task, in particular the tendency of human subjects to persistently choosing a single option when there are multiple equally good options. Here, we propose an alternative explanation of such persistent behavior, by modeling human behavior using a Bayesian hidden Markov model, the Dynamic Belief Model (DBM). We find that DBM captures human choice behavior better than the previously proposed asymmetric RL model. Whereas asymmetric RL attains a measure of optimism by giving better-than-expected outcomes higher learning weights compared to worse-than-expected outcomes, DBM does so by progressively devaluing the unchosen options, thus placing a greater emphasis on *choice history* independent of reward outcome (e.g. an oft-chosen option might continue to be preferred even if it has not been particularly rewarding), which has broadly been shown to underlie sequential effects in a variety of behavioral settings. Moreover, previous work showed that the devaluation of unchosen options in DBM helps to compensate for a default assumption of environmental non-stationarity, thus allowing the decision-maker to both be more adaptive in changing environments and still obtain near-optimal performance in stationary environments. Thus, the current work suggests both a novel rationale and mechanism for persistent behavior in bandit tasks.

## Introduction

Humans frequently overestimate the likelihood of desirable events while underestimating the likelihood of undesirable ones: a phenomenon known as *unrealistic optimism*. For instance, smokers, both former and current ones, underestimate their risk of developing lung cancer and cardiovascular diseases (Masiero, Riva, Oliveri, Fioretti, & Pravettoni, 2016). Likewise, university students overestimate their life expectancy (Clarke, Lovegrove, Williams, & Machperson, 2000), and underestimate possibilities of suffering from heart problems (Green, Grant, Hill, Brizzolara, & Belmont, 2003) and alcoholism (Dillard, Midboe, & Klein, 2009).

One hypothesized mechanism for optimism bias is asymmetrical belief updating (Sharot, Korn, & Dolan, 2011), whereby belief-updating is more influenced by better-than-expected outcomes than by worse-than-expected outcomes. This hypothesis has been elaborated under a reinforcement learning framework, via a modification to the basic Rescorla-Wagner delta-rule learning model (RW) (Rescorla & Wagner, 1972), termed the RW± model (Lefebvre, Lebreton, Meyniel, Bourgeois-Gironde, & Palminteri, 2017). RW± includes two different learning rates, corresponding to updates following positive and negative prediction errors, respectively. It was shown that RW± better captures human behavior in a two-armed bandit task than RW (Lefebvre et al., 2017). Moreover, subjects whose behavior was better explained by RW± than basic RW showed significantly higher learning rates for positive prediction errors, consistent with the suggestion that unrealistic optimism arises from diminished coding of undesirable information (Sharot, 2011).

Here, we propose an alternative explanation of ”optimistic” behavior in the bandit task. We recently found that humans underestimate reward rate of unchosen options in the bandit task (Guo & Yu, 2018), which would encourage sticking with a recently favored option just as asymmetric updating does, as the positive outcomes are amplified. However, unlike the asymmetric RL account, which only biases belief updating and choices based on reward outcome (positive or negative), underestimation of unchosen options leads to their increasing devaluation over time, thus allowing choice history to bias future choices in addition to reward history. For example, an oft-chosen option might continue to be favored even if it has not been particularly rewarding (because the very fact of having chosen it often makes the alternatives appear less inviting). This form of choice-induced bias would be consistent with a broad literature in the study of sequential effects, which has found that choice history biases humans to repeatedly chose a previously chosen option, under a variety of (non-bandit) behavioral settings (Soetens, C, & Hueting, 1985; Wilder, Jones, & Mozer, 2009; Jones, T, Mozer, & Wilder, 2013; Urai, de Gee, Tsetsos, & Donner, 2019).

In this work, we model human bandit choice behavior using a Bayesian hidden Markov model, the Dynamic Belief Model (DBM) (Yu & Cohen, 2009), previously shown to be a good candidate for capturing behavioral data in the multi-armed bandit task (Zhang & Yu, 2013; Guo & Yu, 2018). DBM assumes the reward distribution can undergo change-point dynamics, i.e. occasional re-sampled from a prior distribution (Yu & Cohen, 2009), and thus updates the reward rate estimate by exponentially forgetting past observations (like RW), but in addition persistently injecting a constant prior bias into the estimate of all the arms in every trial (Ryali, Reddy, & Yu, 2018). In particular, this prior bias affects unchosen options much more than the chosen option, whereby the prior pessimism is countered by empirical observations. It was suggested that this relative devaluation of unchosen options helps the subject to counter an excessive exploratory tendency induced by the non-stationarity assumption of DBM, resulting in overall more rewards earned (Guo & Yu, 2018). DBM was found to better account for human behavior than other learning models including RW, but RW± was not tested (Zhang & Yu, 2013; Guo & Yu, 2018).

We re-analyze data from the main experiment of a recent paper (Lefebvre et al., 2017) that found RW± accounts for human behavior better than basic RW. We expect RW± and DBM to both capture human unrealistic optimism to some extent. However, the underlying mechanisms they entail are different: RW± characterizes unrealistic optimism as asymmetrically reduced learning rate for negative prediction errors (or equivalently, asymmetrically higher learning rate for positive prediction errors), while DBM captures unrealistic optimism as a result of prior reward rate underestimation and devaluation of unchosen options. Investigating the differences between the two hypotheses will therefore offer valuable insights into the computational mechanism that underpins unrealistic optimism, and help guide analysis of relevant neural data to unveil the neural basis of unrealistic optimism.

## Results

We re-analyze human behavioral data from a two-armed bandit task (Lefebvre et al., 2017) (see details in Methods). We fit the behavioral data with two learning models, RW± (Lefebvre et al., 2017) and the Dynamic Belief Model (DBM) (Yu & Cohen, 2009; Zhang & Yu, 2013; Guo & Yu, 2018). The RW± model uses two different learning rates, ε^+^ and ε^−^, for positive and negative prediction errors, respectively. The generative model of DBM assumes the reward rates to undergo discrete, unsignaled changes (change-point dynamics): with probability α, the reward rate of an option stays the same, and with probability 1−α, it is re-sampled from a general prior distribution. On each trial, DBM updates the posterior reward rate distribution of the chosen arm using Bayes’ Rule; on the next trial, it updates the predictive prior distribution of the chosen option by mixing its posterior distribution on the last trial with the general prior distribution, with the mixing proportion being determined by α (see Methods). Separately, we have shown that the mean reward rate of DBM is well-approximated by a reinforcement-learning-like rule that mixes Rescorla-Wagner delta rule (RW) with a persistent prior bias *p*_0_, whose value is the prior mean of DBM (Ryali et al., 2018). For the *unchosen arm*, there is no observation, and therefore no Bayes’ Rule updating of the reward rate distribution; however, the assumption of change-point non-stationarity still applies, and the predictive prior is repeatedly mixed with the prior distribution. This leads the estimated reward rate of an unchosen arm to evolve exponentially toward the prior mean (see Methods). For both RW± and DBM, we consider two decision policies: softmax and ε-greedy (see Methods). Given two bandit arms, the essential difference between softmax and ε-greedy is that the former allocates choice probability between the two options depending on how similar their estimated reward rates are (more similar reward rates would lead to more similar choice probabilities), while the latter only cares about which one has the higher reward rate and chooses that with a fixed probability 1−ε (and the other option with probability ε).

### Model Comparison

**Figure 1:**
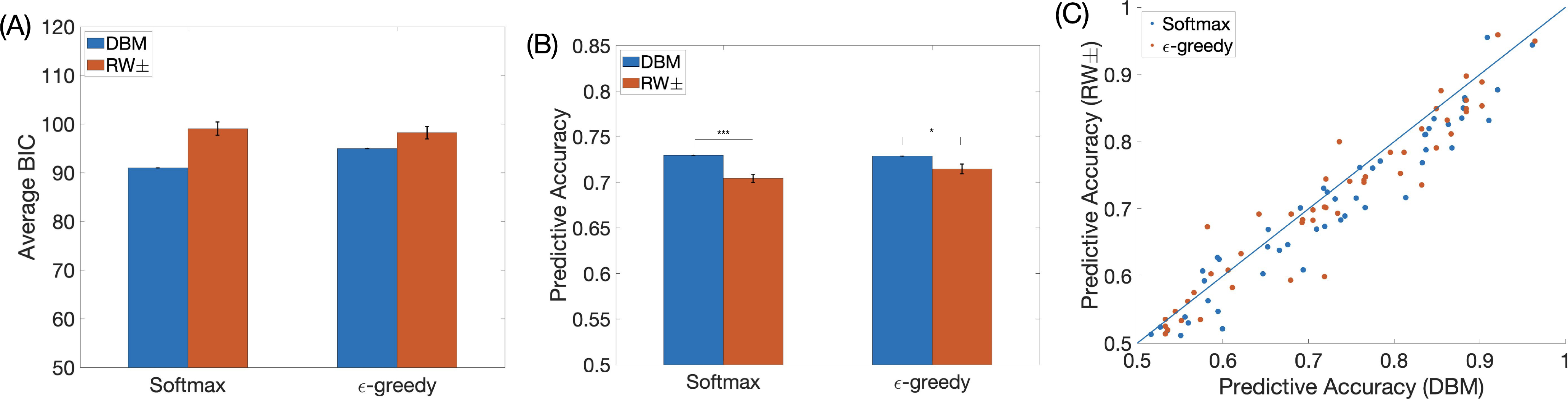
Model comparison. (A) BIC of DBM versus RW±, for both softmax and ε-greedy decision policies. Error bars: s.e.m. of BIC having subtracted out BIC for DBM for each subject, thus error bar for DBM is 0. (B) Average predictive accuracy of DBM versus RW±, for both softmax and ε-greedy decision policies. Chance predictive accuracy is 0.5. Error bars: s.e.m. of individually subtractively normalized predictive accuracy, analogous to (A). (C) Predictive accuracy of DBM versus RW± at the individual level.

We first compare DBM and RW± in terms of how well they capture human behavioral data. Note that DBM and RW± both have two parameters: DBM – the stability parameter and the prior mean; RW± – the positive and negative learning rates. Model parameters are estimated using maximum likelihood estimation. We then compare the four models (2 learning models, 2 decision policies) via two methods: BIC scores (lower the better) and predictive accuracy (higher the better). Given the equal number of free parameters (resulting in a constant offset for both learning models), differences in BIC scores directly reflect differences in log likelihood of the training data (Figure 1A). Once fitted, both softmax and ε-greedy assign a predictive distribution over the options. The predictive accuracy of a model is the probability that the subject and the model choose the same option, which can be approximated empirically as the likelihood the model assigns to the subject’s chosen option averaged over trials. In other words, BIC compares the average of the *log likelihood* the models assign to subjects’ chosen options, while predictive accuracy compares the average of the *likelihood* the models assign to subjects’ chosen options.

We find that DBM accounts for subjects’ choice data better, in comparison to RW±, both in terms of BIC (Figure 1A) and predictive accuracy (Figure 1B), whether we use a softmax decision policy or ε-greedy. Numerically, the BIC scores for DBM and RW±, coupled with softmax, are 91.02 (s.e.m. = 4.70) and 99.06 (s.e.m. = 4.26), respectively (note that RW± was shown in Lefebvre et al. (2017) to have lower BIC than basic RW on the same data). The average BIC scores for DBM and RW±, coupled with ε-greedy, are 95.00 (s.e.m. = 4.34) and 98.23 (s.e.m. = 4.15). The predictive accuracy for DBM and RW±, coupled with softmax, are 72.97% (s.e.m. = 1.79% ) and 70.44% (s.e.m. = 1.72%). The predictive accuracy for DBM and RW±, coupled with ε-greedy, are 72.88% (s.e.m. = 1.80%) and 71.48% (s.e.m. = 1.76%). The difference in predictive accuracy is significant (paired *t*-test: t(48) = 5.52, p *<* 0.001 (softmax); t(48) = 2.66 (ε-greedy), p = 0.011).

At the individual level, more subjects are better fit by DBM than RW± (Figure 1C). Concretely, DBM has better (lower) BIC score than RW± for 40 out of 49 subjects (softmax) or 33 out of 49 subjects (ε-greedy); DBM also has better (higher) predictive accuracy for 39 out of 49 subjects (softmax) or 33 out of 49 subjects (ε-greedy).

For this data set, there is no statistical difference in softmax and *epsilon*-greedy in their respective predictive accuracy in capturing human choice behavior (paired t-test, DBM: *p* = 0.8, RW±: *p* = 0.1). Given this lack of difference, we concentrate only on the softmax policy in the remainder of the paper.

**Figure 2:**
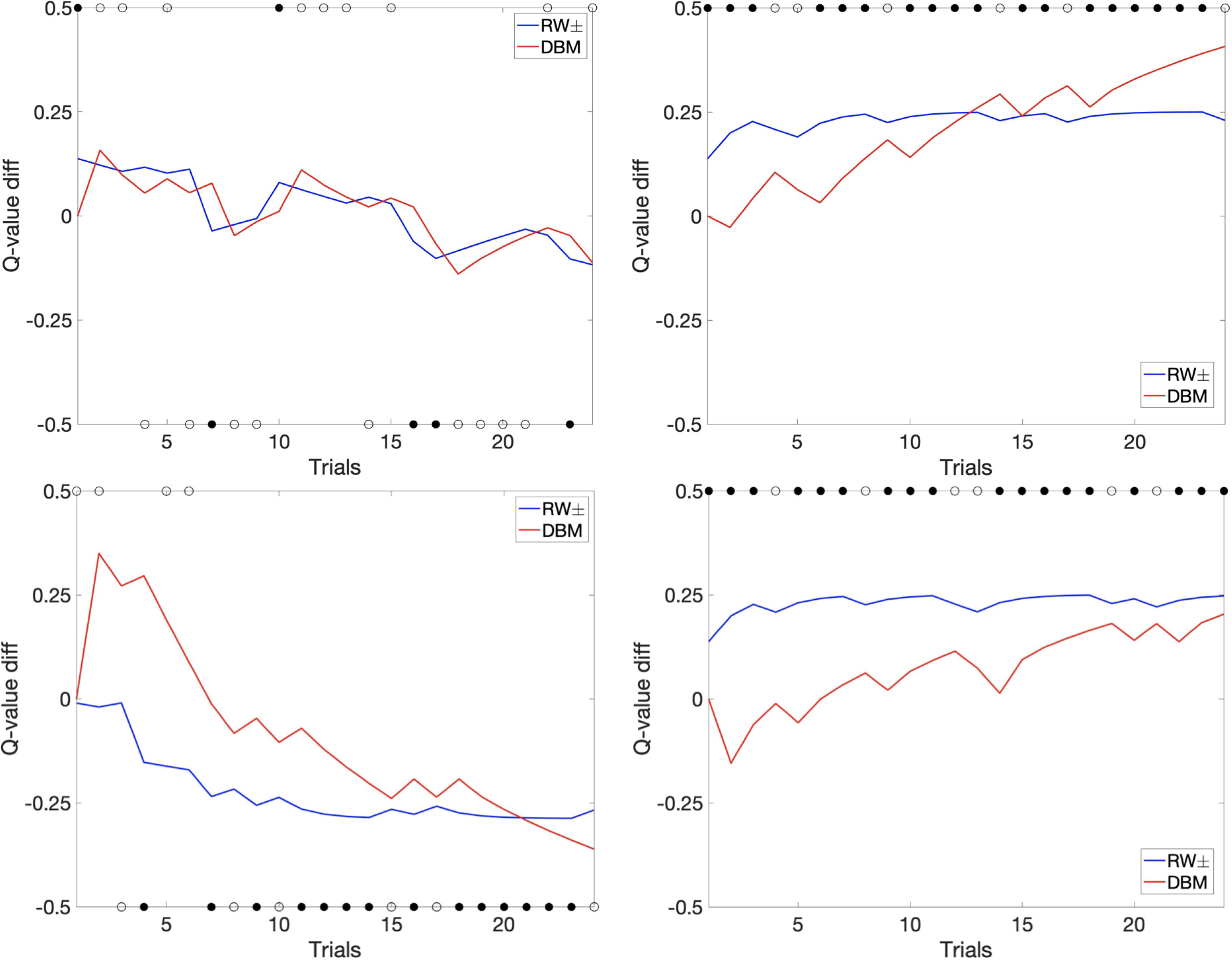
Evolution of differential Q-value (left - right) as a function of trials for an example subject (subject 24). Circles indicate the subject’s actual choice (.5 = left, −.5 = right). More positive Q-value difference means greater model-predicted probability of choosing left arm. Filled circles correspond to reward, and hollow circles correspond to no reward. Top left: 25/25%; Top right: 75/25%; Bottom left: 25/75%; Bottom right: 75/75%. The four pairs were randomly interleaved in their presentation.

To get a better sense for how DBM better accounts for subjects’ behavioral choices than RW±, we consider example sequences of actual choices and outcomes for one example subject, and see how the two models behave differently. We denote the estimated reward rate of the left option as *Q*_left_, and the right option as *Q*_right_, then their difference drives the choices (in the softmax decision policy). As Figure 2 shows, this particular subject has a strong tendency to stick with one choice, whether in the unequal condition (75/25, 25/75) or in the neutral condition (75/75). The only way for RW± to capture this behavior is to make the learning rate very small (ε^−^ = 0.04), such that the Q value stays at a somewhat favorable value in an asymptotically stable manner, as long as the subject continues to exclusive prefer an option; this also has the unfortunate consequence that the human-preferred option is never assigned a very positive Q-value (for this subject, the Q-value difference in RW± never exceeds 0.25 toward the more preferred option). DBM, on the other hand, due to its action-based bias (devaluation of unchosen options), is able to decouple the learning rate for reward estimation from a tendency to persistently favor an option. As such, it allows the estimated reward rate to continue to increase for the truly more rewarding option (the 75% option in the 75/25 and 25/75 conditions), eventually assigning higher likelihood (predictive accuracy) and log likelihood to the better and persistently preferred option. One prediction based on this observation is that, had the number of trials per game (per pair) been larger, DBM would have gained even more advantage over RW ± in capturing human choice behavior, as it is later on in the game that DBM’s ability to assign increasingly higher Q value to the better option becomes more clearly advantageous. Another way of seeing why DBM has higher predictive accuracy of human choice behavior is in the noise parameter of the estimated decision policy. DBM combined with softmax results in a higher inverse temperature parameter (greater predictive precision) than does RW± (DBM: mean 17.07, s.e.m. = 2.19; RW±: mean 10.52, s.e.m. = 1.43), indicating that subjects’ choices are more deterministic (more accurately predicted) relative to DBM’s predictions than RW±’s predictions.

### Model Parameter Analysis

Given that previously it was found that subjects both underestimate reward rates of unseen arms in self-report and in fitted prior mean (Guo & Yu, 2018), we also examine the fitted prior mean in this data set. While the true prior mean of the reward rates used in the experiment is 0.5, we find that the estimated prior mean has a mean value of 0.19 across subjects (s.e.m. = 0.03) and is significantly lower than the true mean 0.5 (*t*-test: t(48) = 7.71, *p* < 0.001). In other words, we replicate the previous finding that subjects significantly under-estimate prior reward rate in the environment (Guo & Yu, 2018).

In addition, we find that the fitted stability parameter α is on average 0.92 (s.e.m. = 0.023) across 49 subjects. It implies that subjects behave as if they believe the reward rates to change on average approximately once every 13 trials (expected interval between change points is 1/(1-α)). This fitted α parameter is relatively high compared to previous bandit tasks (Zhang & Yu, 2013; Guo & Yu, 2018), which typically found α to be between 0.7 and 0.8. This may be because that subjects in this task underwent substantial pre-training with the same stimuli and statistics prior to the main experiment (Lefebvre et al., 2017), unlike in previous studies.

### Model Recovery

To assess model identifiability, we generate synthetic choices and outcomes using DBM, and fit both DBM and RW± on the simulated data. As expected, DBM has a lower BIC score on the data generated using DBM (DBM: mean = 55.84, RW±: mean = 109.13; paired *t*-test: t(48) = 7.1078, *p* < 0.001). Moreover, when fitting RW± on the data generated by DBM, the positive learning rate is on average higher than the negative learning rate (mean ε^+^ = 0.3392, mean ε^−^ = 0.1106, paired *t*-test: t(48) = 3.4208, *p* < 0.001). This result indicates that if subjects truly behave like DBM (with under-estimated prior reward rate), model fitting using RW± would recover an asymmetry belief updating effect, as was found in the original study (Lefebvre et al., 2017). Separately, we also generate synthetic data from RW±, and find RW± to have lower BIC (mean = 99.78) than DBM (mean = 123.00).

## Methods

### Data

We re-analyze data from Lefebvre et al. (2017) experiment 1. 50 healthy adult subjects (mean age = 27.1±1.3, 27 males) were recruited to participate in a two-armed, real-valued outcomes bandit task. Each chosen arm led to either a reward (0.5⋹) or nothing (i.e. 0⋹). To model the reward as a Bernoulli sample in DBM, we converted real-valued rewards (i.e. 0⋹/0.5⋹) to binary values: (0/1 respectively). There were 4 fixed pairs of arms (i.e. 4 conditions), with their respective fixed reward rates: 25/25%, 25/75%, 75/25%, and 75/75%. Thus, it was a 2×2 design, varying both general reward availability (high versus low) and asymmetry (equal versus unequal). During the main experiment, each subject was exposed to each pair (condition) 24 times in total, with 4 conditions interleaved – the order of all 96 trials were randomized for each subject. No explicit information regarding reward rates were given to the subjects. Subjects were instructed to earn as much money as possible, and they were told some arms were more rewarding than others, but not how much.

### Model description

We consider two learning models, DBM (Yu & Cohen, 2009; Zhang & Yu, 2013; Guo & Yu, 2018) and RW± (Lefebvre et al., 2017), each coupled with two decision policies, softmax and ε-greedy.

Let $k_{i}^{n}$ denote arm *i* in the *n*th condition, where 1 ≤ *n* ≤ 4, *i* ∈ {1, 2}. Moreover, let $\theta_{k_{i}^{n}}^{t}$ denote the reward rate of arm *i* in the *n*th condition at time *t*, with 1 ≤ *t* ≤ 96. For simplicity, let $k_{i}^{n}\in \{1,2,\ldots ,8\}$, and $k_{i}^{n}=(n-1)*2+i$ (e.g. first arm in condition 1 is 1, second arm in condition 2 is 2.etc). Furthermore, ∀*t.*1 ≤ *t* ≤ 96, let *d*_*t*_ denote the decision at time *t*, *d*_*t*_ ∈ {1,2,…,8}, and *r*_*t*_ denote the reward outcome at time *t*. For DBM, reward data were converted so that *r*_*t*_ ∈ {0, 1} (see below). For RW±, *r*_*t*_ ∈ {0, 0.5}, consistent with the exact monetary reward in the experiment design. Finally, let **D**^*t*^ denote the decision history up to time *t*, and **R**^*t*^ the reward history up to time *t*. i.e. **D**^*t*^ = [*d*_1_,*d*_2_,…,*d*_*t*_] and **R**^*t*^ = [*r*_1_,*r*_2_,…,*r*_*t*_].

### Dynamic belief model (DBM)

DBM assumes the rewards are binary-valued (i.e. 1 = reward, 0 = no reward), following a Bernoulli distribution for each arm. It also assumes the reward rate of each arm to be non-stationary: at a given time point, there is a 1−α probability that the reward rate of an arm will be re-sampled from a prior distribution *p*^0^(θ), and α probability remaining the same as the last encounter:

(1) $$p\left(\theta_{k_{i}^{n}}^{t}=\theta |\theta_{k_{i}^{n}}^{t-1}\right)=(1-\alpha )p^{0}(\theta )+\alpha \delta \left(\theta_{k_{i}^{n}}^{t-1}-\theta \right)$$

where δ(*x*) is the Dirac delta function.

For an arm $k_{i}^{n}$, the predictive reward rate distribution is

(2) $$p\left(\theta_{k_{i}^{n}}^{t}|R^{t-1},D^{t-1}\right)=(1-\alpha )p^{0}(\theta )+\alpha p\left(\theta_{k_{i}^{n}}^{t-1}|R^{t-1},D^{t-1}\right).$$

For the chosen arm, the posterior distribution is updated according to Bayes’ rule:

(3) $$p\left(\theta_{k_{i}^{n}}^{t}|R^{t},D^{t}\right)\propto p\left(R^{t}|\theta_{k_{i}^{n}}^{t}\right)p\left(\theta_{k_{i}^{n}}^{t}|R^{t-1},D^{t-1}\right),\;\text{if}\;d^{t}=k_{i}^{n}.$$

For the unchosen arms (both the unchosen arm that is seen and all the unavailable not seen), the posterior distributions remain the same as the priors, but the predictive distribution will be updated, leading the predictive mean to converge toward the prior mean when an arm has not been chosen for a long time.

### Rescorla-Wagner± model (RW±)

In contrast to a standard Rescorla-Wagner (RW) model, which has a single learning rate (e.g. ε), RW± has two (potentially) different learning rates for positive and negative prediction errors respectively. i.e. ε^+^ for positive prediction errors and ε^−^ for negative prediction errors. In other words,

(4) $${\hat{\theta }}_{k_{i}^{n}}^{t}={\hat{\theta }}_{k_{i}^{n}}^{t-1}+\{\begin{array}{ll} \varepsilon^{+}\left(r_{t}-{\hat{\theta }}_{k_{i}^{n}}^{t-1}\right), & \text{if}\;r_{t}-{\hat{\theta }}_{k_{i}^{n}}^{t-1}>0\\ \varepsilon^{-}\left(r_{t}-{\hat{\theta }}_{k_{i}^{n}}^{t-1}\right), & \text{if}\;r_{t}-{\hat{\theta }}_{k_{i}^{n}}^{t-1}<0 \end{array},$$

where 0 ≤ε^+^,ε^−^ ≤ 1, and $\theta_{k_{i}^{n}}^{0}=\theta^{0}$ for all $k_{i}^{n}$’s. Note that only the chosen arm’s estimated reward rate is updated according to any new observation.

### Softmax Decision Policy

The probability (likelihood) of choosing a particular arm $k_{i}^{n}$ at time *t* is given by

(5) $$p\left(d^{t}=k_{i}^{n}\right)=\frac{e^{b{\hat{\theta }}_{k_{i}^{n}}^{t}}}{e^{b{\hat{\theta }}_{k_{1}^{n}}}+e^{b{\hat{\theta }}_{k_{2}^{n}}}}$$

where *b* is the softmax inverse-temperature parameter.

### ε-greedy Decision Policy

The probability (likelihood) of choosing a particular arm $k_{i}^{n}$ at time *t* is given by

(6) $$p\left(d^{t}=k_{i}^{n}\right)=(1-\varepsilon )*1\left\{{\hat{\theta }}_{k_{i}^{n}}^{t}=max_{j}{\hat{\theta }}_{k_{j}^{n}}^{t}\right\}+\frac{\varepsilon }{K}$$

where ε indicates the probability of exploration, and *K* is the number of available arms in the current condition (in this case, *K* = 2).

### Model Fitting

We fit the models by maximizing total log likelihood, summed over trials, for each subject. We discretize the parameter space to find the setting yielding the highest log likelihood. For DBM, we set the prior weight (*a* + *b*, where *a* and *b* are the parameters in the Beta prior Beta(a,b)) (Zhang, Huang, & Yu, 2014), which is somewhat informative but not too strong a prior bias. We set the initial Q-values for RW± to be 0.5 as in the original paper (Lefebvre et al., 2017): 0.5 is the true generative mean reward rate of all the arms. We fit all other model parameters individually for each subject.

### Model Recovery

We simulate data with best individually fitted parameter for the two models (N=49) under the same setting as the experiment. We simulate the same parameter sets 9 times. The recovered prior mean (Pearson’s correlation test: *r* = 0.8364, *p* < 0.001), α (Pearson’s correlation: *r* = 0.6596, *p* < 0.001), and softmax parameter (Pearson’s correlation: *r* = 0.4704, *p* < 0.001) are all positively and strongly correlated with true parameters.

### Analysis of Side Bias

We consider the possibility that some subjects may exhibit significant side bias, especially since two of the pairs have equal reward rates, and subjects had substantial pre-training with these same stimuli before the main session and may have learned it does not matter which option they choose. To examine a potential side bias, two versions of decision models are compared: one with softmax decision policy, and one with a mixed decision policy, which is a linear combination of softmax and a categorical variable indicating the presence of either a left or a right side bias. i.e.

(7) $$p\left(d^{t}=k_{i}^{n}\right)=\beta \tau +(1-\beta )\frac{e^{{\hat{\theta }}_{i}^{n}\cdot b}}{e^{{\hat{\theta }}_{k_{1}}^{t}\cdot b}+e^{{\hat{\theta }}_{k2}^{t}\cdot b}},$$

where β ∈ [0,1], τ ∈ {τ_*l*_, τ_*r*_}. τ_*l*_ = 1 if the decision is made with a left side bias and 0 otherwise; τ_*r*_ = 1 if there is a right side bias and 0 otherwise. Note when β = 0, the above decision policy is exactly softmax (no side bias). When β = 1, it implies the decision is made only using a side bias and no consideration of reward value at all. We use DBM to predict the estimated reward rate of each arm, and couple it with the two decision policies (softmax and mixed) separately to determine which model offers a better prediction of human behavioral data.

We find there is only one subject (Subject 19 in the original dataset) whose behavior is much better captured by DBM with a left side bias than without, with a fitted β = 0.7 – that is, the subject chooses the left option 70% of the time without learning. A post-hoc analysis reveals that this subject, regardless of the actual reward rates in each condition, quickly narrowed down to the left option despite little or no information about the reward rate of the right option in all conditions. The data associated with this subject are consequently excluded from all analyses.

There are 16 additional subjects in the dataset whose behavior is (slightly) better captured by DBM with the mixed decision policy (τ = τ_*l*_). However, all these subjects have a fitted β less than 0.1, meaning the effect of (left) side bias is relatively minimal. We choose to retain these subjects and employ the pure softmax decision policy, as there any side bias appears to have a small behavioral impact among these subjects.

## Discussion

In this work, we re-analyzed a two-armed bandit task data set previously used to support asymmetric belief updating (greater weight to “reward” than “no reward” outcomes) (Lefebvre et al., 2017), which had been suggested to be a mechanistic source of human optimism bias (Sharot, 2011). We found that the behavioral choice data is actually better accounted for by a Bayesian ideal observer model (DBM), which (incorrectly) assumes environmental non-stationarity (Yu & Cohen, 2009; Zhang & Yu, 2013; Guo & Yu, 2018), and has been shown to capture human behavior in a broad range of behavioral tasks (Yu & Cohen, 2009; Zhang & Yu, 2013; Yu & Huang, 2014; Ma & Yu, 2015; Guo & Yu, 2018). As was reported previously (Guo & Yu, 2018), using DBM, we also found in this data set that subjects increasingly devalue unchosen options. It was previously suggested (Lefebvre et al., 2017) that the striking persistence with which human subjects repeatedly choose the same option, when the two bandit options have equal reward rates, reflects a form of optimism bias (biased estimate that the preferred option is more rewarding). However, our work suggests an alternative explanation, that the bias is not in terms of reward versus no reward, but chosen option versus unchosen option. Because the two are highly correlated in most bandit tasks, as subjects are generally able to find and mostly choose the more rewarding option, the two models make highly correlated predictions. This is reflected in the relatively small effect size we found in terms of the improvement of DBM over RW± in explaining human data. Future work is needed to identify experimental scenarios in which the two kinds of biases would make more distinct predictions.

Separately, we were not able to distinguish two possible types of ”forgetting”: whether to devalue only the 1 available unchosen arm, or also the other 6 unavailable chosen arms, on each trial. Answering this question may have implications of the area of decision neuroscience known as counterfactual learning. We reported data from the version of DBM that mixes the posterior of *all* unchosen arms (7 out of 8 total arms) with the prior distribution at each time step, regardless of whether they are available to the subject or not. An alternative approach is to only update the one unchosen arm available at each time step while keeping reward rate estimations for other unavailable arms constant. We also implemented the alternate version, but did not find any significant statistical difference between them on this data set (results not shown). A larger future study, with more participants, more trials, and more arms, would be helpful for clarifying this point.

Another limitation of this study is that the task only involves two arms, which makes it hard to interpret the exact rationale of subjects’ choice. For example, when they switch from the left option to the right option, is it because they no longer liked the left option or especially wanted to try the right option? This question cannot be answered without at least three arms. A related issue is that we found softmax and ε-greedy to be statistically indistinguishable in explaining choice behavior. With more arms, the two policies would make more differentiated choices, and thus be more easily teased apart.

An obvious question that arises from our work is why subjects should assume non-stationarity by default and underestimate reward rate. As was previously argued (Yu & Cohen, 2009; Zhang & Yu, 2013), the assumption of non-stationarity allows subjects to readily adapt to changing environments outside this particular, synthetic experimental setting. On the other hand, this non-stationary assumption tends to push the decision maker to give up on previously good options too quickly due to a chance bad outcome (Guo & Yu, 2018); devaluation of unchosen options (by under-estimating reward rates in the prior) mitigates this tendency and improves overall performance in a fixed environment (Guo & Yu, 2019). Thus, the current work suggests both a new rationale and mechanism for a form of excessive optimism in humans: humans assume environmental non-stationarity by default to improve adaptability, but this causes giving up on good options too quickly in noisy stationary environments; excessive optimism about the chosen option (maintained by devaluing unchosen options) discourages the decision maker from giving up on good options too quickly.
